# The role of long non-coding RNAs in the regulation of pancreatic beta cell identity

**DOI:** 10.1042/BST20210207

**Published:** 2021-09-28

**Authors:** Maya E. Wilson, Timothy J. Pullen

**Affiliations:** Department of Diabetes, School of Life Course Sciences, King's College London, London, U.K.

**Keywords:** cell identity, insulin, islet, long non-coding RNA (lncRNA), pancreatic beta cell

## Abstract

Type 2 diabetes (T2D) is a widespread disease affecting millions in every continental population. Pancreatic β-cells are central to the regulation of circulating glucose, but failure in the maintenance of their mass and/or functional identity leads to T2D. Long non-coding RNAs (lncRNAs) represent a relatively understudied class of transcripts which growing evidence implicates in diabetes pathogenesis. T2D-associated single nucleotide polymorphisms (SNPs) have been identified in lncRNA loci, although these appear to function primarily through regulating β-cell proliferation. In the last decade, over 1100 lncRNAs have been catalogued in islets and the roles of a few have been further investigated, definitively linking them to β-cell function. These studies show that lncRNAs can be developmentally regulated and show highly tissue-specific expression. lncRNAs regulate neighbouring β-cell-specific transcription factor expression, with knockdown or overexpression of lncRNAs impacting a network of other key genes and pathways. Finally, gene expression analysis in studies of diabetic models have uncovered a number of lncRNAs with roles in β-cell function. A deeper understanding of these lncRNA roles in maintaining β-cell identity, and its deterioration, is required to fully appreciate the β-cell molecular network and to advance novel diabetes treatments.

## Introduction

Type 2 diabetes (T2D) is a worldwide epidemic affecting over 400 million people [1]. A combination of genetic and environmental risk factors results in a mismatch between the insulin released and the insulin required to correctly regulate blood glucose levels. The resulting sustained hyperglycaemia causes numerous complications including neuropathy, nephropathy and cardiovascular disease which together impose a serious burden of morbidity and mortality.

In healthy individuals, pancreatic β-cells regulate blood glucose levels by the tightly controlled secretion of insulin in response to glucose. To achieve this, β-cells have a highly specialised metabolic system whereby the glucose entering glycolysis is directly proportional to the extracellular concentration. Glucose carbons are then efficiently transported to the mitochondria for oxidative phosphorylation to generate ATP which in turn triggers insulin release [2]. This requires the expression of a group of genes involved in glucose sensing (including *SLC2A2* (*GLUT2*), *GCK*) and insulin production and processing (including *INS, PCSK1*). Alternative metabolic pathways which could reduce the efficiency of this process are shut off, including the conversion of pyruvate to lactate and subsequent export from the cell (encoded by *LDHA* and *SLC16A1*) [3]. The unique complement of transcription factors essential for maintaining this includes *PDX1, MAFA, NEUROD1, NKX6-1* and *PAX6* [4]. The ability of β-cells to sense and release sufficient insulin is therefore critically dependent on this pattern of gene expression, which defines mature β-cell identity

In the early stages of T2D, the β-cells’ inability to fully compensate for increased insulin resistance results in impaired glucose tolerance. The increased demand for insulin coupled with the resulting hyperglycaemia progressively damage the β-cells until their decline in function causes overt T2D. Over the last ten years, it has become apparent that a loss of β-cell identity contributes to their functional decline. This is characterised by the reduced expression of key β-cell transcription factors and the networks of proteins they regulate, as well as increased expression of normally disallowed genes [5]. The maintenance of β-cell identity is critical for glucose homeostasis and understanding how identity is maintained is important to understanding the pathology of T2D.

Long non-coding RNAs (lncRNAs) are transcripts >200 nucleotides long that are typically post-transcriptionally processed but do not encode proteins [6]. They are often enriched in the nucleus and regulate gene expression via a range of mechanisms [6]. lncRNAs differ from some classes of short non-coding RNAs, such as microRNAs (miRNA) which have a more clearly defined mechanism of action. miRNAs are processed from longer transcripts, then associate with AGO2 to target mRNAs for translational inhibition and/or degradation via complementary base pairing [7]. This allows miRNA targets to be predicted bioinformatically. Since lncRNAs are a heterogenous group of transcripts acting via different mechanisms, and the nature of the interactions with other transcripts and proteins are less well known, lncRNA target prediction has generally been much less successful.

While lncRNAs are generally expressed at lower levels than their protein-coding counterparts, their expression patterns are more tissue-specific meaning they are well placed to regulate cell identity [8–10]. In this mini-review, we will discuss the role that lncRNAs play in regulating β-cell identity and the implications for T2D.

## lncRNAs: history, mechanisms and conservation

In the early 1990s, a small number of RNA molecules were identified which were transcribed by RNA polymerase II, spliced and polyadenylated like mRNAs, but appeared to lack open reading frames. These included *H19*, expressed from the imprinted *Igf2* locus [11] and *XIST*, from the X chromosome inactivation centre [12]. Deletion experiments identified that *Xist* was required for X chromosome inactivation [13] and later studies showed that the lncRNA achieves this by recruiting Polycomb Repressive Complex 1 (PRC1) [14]. *Airn*, expressed from the imprinted *Igf2r* locus is a long unspliced transcript whose expression is required for silencing three other imprinted genes in this cluster (*Igf2r*, *Slc22a2* and *Slc22a3*) [15]. These firmly established a role for long non-coding RNAs (lncRNAs) in regulating gene expression.

Technological advances, first in the form of tiling arrays and later high throughput sequencing, identified many more non-protein-coding transcripts. These included *HOTAIR*, a lncRNA expressed from the *HOXC* locus on chromosome 12, which interacted with Polycomb Repressive Complex 2 (PRC2) to mediate histone H3 lysine-27 (H3K27) trimethylation and gene silencing at the *HOXD* locus on chromosome 2 [16]. This demonstrates that lncRNAs can regulate nearby genes both in *cis*, as well as across much larger distances, in *trans*.

By 2012, the GENCODE project had identified over 9000 human lncRNA genes [8]. Whether many of these lncRNAs are functional molecules or transcriptional by-products became a point of debate, partly due to their low inter-species conservation and low expression levels [17]. However, investigation of individual lncRNAs has continued to reveal their functional effects and expanded the known mechanisms by which they function. *H19* is implicated in tumorigenesis and modulates miRNAs both positively and negatively. The lncRNA is processed to release the growth-suppressing miRNA miR-675 [18]. It also binds to and sequesters several other miRNAs, thus acting as a miRNA ‘sponge’ to down-regulate their action [19]. *HOTAIR* has a range of *trans* inhibitory functions spanning the nucleus and cytoplasm, including recruitment of histone demethylases for gene suppression and ubiquitin ligase recruitment for protein degradation [20]. These mechanisms are non-mutually exclusive as lncRNAs may have diverse cellular roles [6].

While their sequences are less conserved than protein-coding sequences, lncRNAs are significantly more conserved than random intergenic regions [21]. lncRNA function is facilitated by the ability to form secondary structures that enables their interaction with nucleic acids and proteins. lncRNAs also interact with mRNAs to alter their stability or miRNAs to sequester them based on nucleotide sequence complementarity. In both cases, lncRNA functionality is less dependent on precise nucleotide sequence as compared with amino acid sequence determining protein function [22]. Furthermore, promoter regions of lncRNA genes have been shown to contain transcription factor binding sites and generally contain similar levels of sequence homology to protein-coding gene promoters [8]. These regions can be used to identify orthologous lncRNA genes between species [23].

## lncRNAs genetically associated with type 2 diabetes

The first clear evidence of lncRNA involvement in β-cell function came from genetic association. It became apparent from early genome-wide association studies (GWAS) that most T2D-associated SNPs occurred in non-protein-coding regions of the genome [24]. While variants in protein-coding regions often result in an amino acid substitution or protein termination, the molecular effect of variants in non-protein-coding regions can be harder to determine. Several T2D-associated SNPs were identified in loci expressing lncRNAs including *CDKN2B-AS1* (*ANRIL*) [25–27] and *KCNQ1OT1* [28,29]. Indeed, the *CDKN2B-AS1* locus cropped up in so many diverse studies of susceptibility to cancer and metabolic disease that it was described as a ‘GWAS hotspot’ [30].

**Figure 1.**
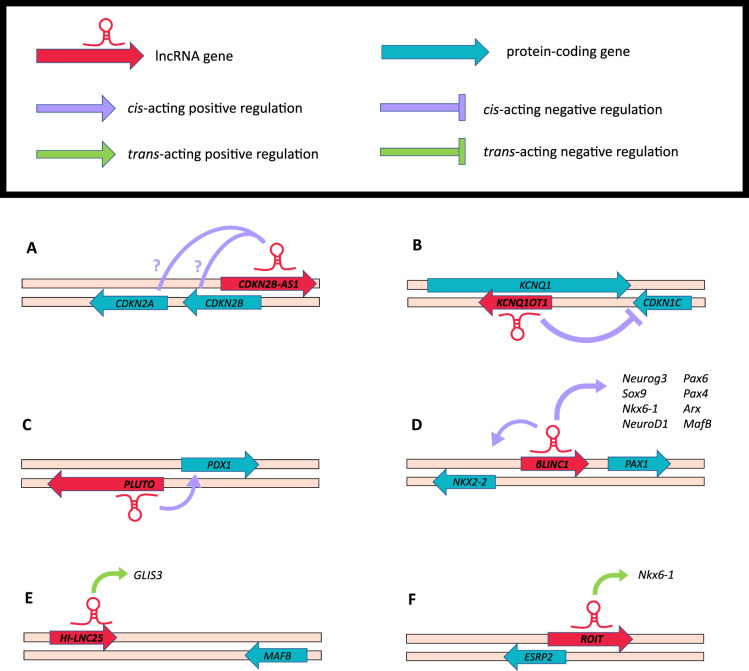
Genomic positioning and directional regulation of lncRNA genes in relation to nearby β-cell genes. (**A**) *CDKN2B-AS1* (*ANRIL*) mechanism of action remains unclear, as it has potential PRC1/2 binding potential, but is also positively correlated with *CDKN2A/B*. (**B**) *KCNQ1OT1* negatively regulates its neighbour *CDKN1C* to maintain β-cell proliferation. (**C**) *PLUTO* positively regulates *PDX1* by enhancing its promoter-enhancer cluster contacts. (**D**) *βlinc1* has wide-spread cis effects on local gene expression to maintain β-cell function, positively regulating *Nkx2-2*, *Neurog3*, *Sox9*, *Nkx6-1*, *NeuroD1*, *Pax6*, *Pax4*, *Arx* and *MafB*. (**E**) *HI-LNC25* has a *trans*-acting effect on *GLIS3*, located on another chromosome. (**F**) *ROIT* neighbours *ESRP2* but has *trans* effects on *Nkx6-1*.

The *CDKN2A/B* locus contains two protein-coding genes encoding the three well-studied cyclin-dependent kinase inhibitors p16INK4A, p15INK4B and p14ARF, and a lncRNA gene *CDKN2B-AS1* (Figure 1A). The importance of this locus to the regulation of cellular proliferation explains the links to cancer and suggests dysregulation of β-cell proliferation may cause the increased T2D susceptibility. However, disentangling the effects of the various SNPs in this complex locus is not straightforward. The T2D-associated SNPs fall within the lncRNA gene and, at least in peripheral blood samples are more closely associated with the lncRNA expression than the protein-coding genes [30,31]. However, the regulation of this locus differs between cell types and the difficulty in accessing human islet tissue and the lack of conserved lncRNAs in the mouse genome means relatively few studies have directly investigated the function of *CDKN2B-AS1* in β-cells. The best evidence for a role in β-cells is that T2D-associated SNPs increased expression of *CDKN2B-AS1*, and while they did not affect insulin secretion, they were associated with impaired β-cell proliferation [32]. *CDKN2B-AS1* can down-regulate neighbouring genes by recruiting PRC1 and PRC2 components [33]. However, at least in islet samples, the expression of *CDKN2B-AS1* and the transcripts encoding p14 and p16 are strongly positively correlated implying that coregulated expression outweighs any inhibition of the other genes by the lncRNA [32]. *CDKN2B-AS1* has also been shown to regulate gene expression on other chromosomes, including down-regulating *KLF2* to regulate apoptosis in cancers [34,35]. Therefore, while there is evidence for a role for *CDKN2B-AS1* in regulating β-cell proliferation and diabetes susceptibility, the mechanism by which it achieves this remains unclear. The possibility that SNPs at this locus affect diabetes susceptibility through effects in other tissues can also not be excluded [36].

The *KCNQ1* locus is another region that is central to the regulation of cellular proliferation and associated with T2D susceptibility. It is an imprinted region which expresses a lncRNA, *KCNQ1OT1*, orientated antisense to the *KCNQ1* gene which encodes a voltage-gated potassium channel subunit (Figure 1B). *KCNQ1* knockout in mice and knockdown in human islets appears to have no effect on glucose tolerance and insulin secretion, making it very unlikely to affect T2D susceptibility [37]. The locus also contains the *CDKN1C* gene encoding P57KIP2, an inhibitor of cyclin-dependent kinases and known regulator of β-cell proliferation [38]. *KCNQ1OT1* interacts with G9a and PRC2 to mediate histone methylation and down-regulation of *CDKN1C* and *SLC22A18* [39]. The SNPs at this locus are hypothesised to mediate diabetes susceptibility by disrupting the expression of *KCNQ1OT1*, altering the imprinting within this gene cluster and leading to the overexpression of *CDKN1C* and impaired β-cell proliferation [37].

Another imprinted locus regulating cellular proliferation is the *H19*/*IGF2* locus, which both expresses a lncRNA and has more recently been genetically associated with T2D susceptibility [40]. *H19* is an intergenic lncRNA that has been implicated in β-cell mass expansion during neonatal development. Adult β-cells have limited proliferative capacity as compared with neonatal β-cells which are characterised by ∼300 times greater *H19* expression. Silencing *H19* in neonatal rat β-cells reduced their proliferation, whereas overexpression increased proliferation of adult β-cells via the PI3K–Akt pathway. *H19* was also significantly increased in *ob/ob* and *db/db* diabetic mouse models with increased β-cell mass, supporting the role of *H19* in β-cell mass expansion [41].

It is interesting to note that these lncRNAs appear to influence T2D susceptibility through impaired proliferation causing an insufficiency in β-cell mass rather than via a loss of β-cell identity. Since the early GWA studies, many more β-cell lncRNAs have been identified and in some cases functionally characterised. The most comprehensive catalogue of lncRNAs expressed in human β-cells identified 1128, many of which were conserved in mouse [21]. This catalogue has proved a vital resource for subsequent studies.

## β-cell transcription factors and lncRNAs

The key β-cell transcription factors, including PDX1, MAFA, NEUROD1 and PAX6, are responsible for regulating the expression of genes involved in insulin synthesis and GSIS, and are thus central to regulating β-cell identity [4]. Given the potential for lncRNAs to regulate expression of nearby genes, β-cell lncRNAs located near β-cell transcription factor genes have often been selected to identify novel regulators of β-cell identity. These studies have provided good evidence that lncRNAs and β-cell transcription factors can be co-regulated such that if the expression of a lncRNA is altered, its neighbouring protein-coding counterpart will also be altered [42]. For example, expression of the lncRNA *PLUTO* is highly correlated with its neighbour *PDX1* (Figure 1C). Knockdown of *PLUTO* reduced *PDX1* expression and resulted in a similar transcriptional profile to that of a *PDX1* knockdown. Chromatin conformation capture assays revealed that *PLUTO* positively regulates *PDX1* by enhancing contacts between its promoter and an upstream enhancer cluster [42] demonstrating that *PLUTO* regulates gene expression by modifying the 3D chromosomal structure.

*βlinc1* is another lncRNA which appears to regulate a nearby transcription factor gene. The *βlinc1* gene is located between *NKX2-2* and *PAX1* in humans and mice (Figure 1D) and its expression is highly enriched in β-cells [43]. *βlinc1* knockdown in mouse insulinoma cells (MIN6) resulted in down-regulation of *Nkx2-2*, indicating the lncRNA is a positive regulator of *Nkx2-2* expression, although the mechanism is unknown. *βlinc1* knockout mice were glucose intolerant due to an alteration in the ratio of insulin-and somatostatin-producing cells that originated during pancreatic development, as evidence for the role of *βlinc1* in maintaining β-cell identity. Both hetero- and homozygous *βlinc1* knockouts resulted in dysregulated expression of mature functional β-cell genes including *Neurog3*, *Sox9, Nkx6-1, NeuroD1, Pax6, Pax4, Arx* and *MafB*. Many dysregulated genes were found within a ∼55Mb radius of *βlinc1* on chromosome 2, indicating a *cis*-acting role of *βlinc1* in β-cell development and identity [43].

In contrast with the previous two examples, the *PAX6* locus contains a lncRNA gene which appears to be a negative regulator of β-cell identity. *Pax6os1* (mouse)/*PAX6-AS1* (human) is orientated antisense to *PAX6* and displays increased expression in islets from donors with T2D and diabetic mouse models. *Pax6os1* knockdown in MIN6 and CRISPR-mediated knockout in a human β-cell line (EndoC-βH1) resulted in increased expression of *PAX6* and several other β-cell genes including *INS* and *SLC2A2* (*GLUT2*) [44]. However, these effects were not observed in a *Pax6os1* null mouse, possibly because the deletion of the lncRNA promoter and first exon also removed regulatory sequences for *Pax6*.

While many lncRNAs expressed from β-cell transcription factor loci are involved in regulating the expression of nearby genes, some appear to regulate gene expression on other chromosomes*. HI-LNC25* (*LINC01370*) neighbours *MAFB* on chromosome 20 (Figure 1E), yet knockdown of this lncRNA produced no effect on *MAFB* expression while decreasing expression of *GLIS3*, located on chromosome 9 [21]. *GLIS3* encodes a transcription factor important for both β-cell development [45] and insulin transcription [46]. While *HI-LNC25* depletion had no significant effect on GSIS, it was enriched in human islets compared with embryonic pancreas suggesting a developmental role for this lncRNA [21]. Similarly, lncRNA *ROIT* (*1810019D21Rik*) has a role in glucose homeostasis and insulin transcription. *ROIT* regulates expression of the neighbouring *ESRP2* gene (Figure 1F), but also positively regulates *NKX6-1 in trans* by inhibiting DNMT3A-mediated DNA methylation of its promoter [47]. It is therefore clear that lncRNAs expressed from transcription factor loci in β-cells are involved in both local and distant regulation of gene expression underlying β-cell identity.

## lncRNA expression levels in diabetic models

Expression changes in animal models of diabetes have been used to identify lncRNAs regulating β-cell identity. *βlinc2* and *βlinc3* are highly islet- and β-cell-enriched, with *βlinc2* being significantly up-regulated in high-fat diet and *db/db* mice, whereas *βlinc3* tended to be down-regulated in these groups [48]. *βlinc3* was also significantly reduced in islet donors with T2D and negatively correlated with both BMI and HbA1c. *βlinc2* overexpression and *βlinc3* down-regulation both increased apoptosis of MIN6 cells and primary mouse islets yet had no clear effect on insulin synthesis or secretion. Preliminary investigation into *βlinc2* indicated it may stimulate apoptosis by shuttling NF-κB into the nucleus [48].

Two further lncRNAs, *Tug1* and *Meg3,* were singled out because of their glucose-regulated expression. *Tug1* is pancreas-enriched and its expression was reduced in two β-cell lines exposed to increasing glucose concentrations [49]. *In vivo* siRNA-knockdown of *Tug1* resulted in reduced cell proliferation, increased apoptotic caspases and decreased anti-apoptotic *Bcl2 in vitro* and *in vivo*. This was accompanied by reduced GSIS, higher blood glucose levels and decreased expression of β-cell genes *Pdx1*, *NeuroD1*, *MafA* and *Slc2a2* [49], clearly demonstrating that *Tug1* reinforces β-cell identity.

In contrast with *Tug1*, *Meg3* was up-regulated in response to increasing concentrations of glucose in MIN6 cells, correlating well with levels of *Ins2* [50]. siRNA knockdown of *Meg3* led to impaired glucose tolerance, decreased insulin content and insulin secretion during a glucose challenge. Dysregulated *Meg3* also resulted in lowered *Pdx1* and *MafA* expression but had no significant effect on *NeuroD1* or *Nkx6-1*. Like *Tug1*, *Meg3* knockdown inhibited cell growth due to increased apoptosis [50]. Mechanistically, *Meg3* has been shown to interact with PRC2 and form RNA-DNA triplex structures at specific genomic sites, facilitating chromatin silencing in a breast carcinoma cell line [51]. Similarly, RNA immunoprecipitation in MIN6 cells found *Meg3* was associated with the PRC2 component, EZH2. Together they increase *MafA* expression by down-regulating three repressive transcription factors Rad21, Smc3 and Sin3a [52]. These studies have made use of mouse models and cell lines to emphasise the functions of key lncRNAs in β-cell transcriptional regulation, however further studies highlighting the roles of these lncRNAs in humans is required.

The links between lncRNAs and T2D have also been investigated by identifying changes in circulating lncRNAs associated with diabetes. As an example, *lncRNA-p3134* was significantly increased in serum and specifically enriched in the circulating exosomes of humans with diabetes and mouse models. Overexpression of this lncRNA resulted in increased expression of β-cell-specific genes such as *Pdx1*, *MafA* and *Slc2a2*, leading to increased glucose-stimulated insulin secretion (GSIS) via activation of the PI3K/Akt/mTOR pathway. *lncRNA-p3134* overexpression also provided a protective effect against glucotoxicity-mediated apoptosis, thereby maintaining β-cell mass for a sufficient insulin secretory response [53].

In contrast with *lncRNA-p3134*, the lncRNA *GAS5* shows significantly reduced expression levels that correlate with HbA1c and T2D in humans, highlighting its potential use as a serum biomarker [54,55]. siRNA knockdown of *Gas5* in MIN6 cells and mouse islets impaired expression of insulin mRNA and GSIS, as well as reducing expression of β-cell genes *Pdx1*, *MafA*, *NeuroD1* and *Slc2a2*. In contrast, *Gas5* overexpression improved cell viability, GSIS and insulin content [54]. Further investigation into *Gas5* demonstrated a role in miRNA sequestration, as expression levels of three miRNAs with roles in insulin signalling, secretion and resistance (miR-29a-3p, miR-96-3p, and miR-208a-3p) were inversely correlated with *Gas5* levels during *Gas5* overexpression and knockdown. *Gas5* has conserved binding sites for these miRNAs and pulldown assays confirmed their interaction as evidence for its mechanism for maintaining β-cell identity [54]. Another mechanism by which *Gas5* can act is by repressing the glucocorticoid receptor (GR) through competitively inhibiting the DNA binding motif [56]. Glucocorticoids (GC) inhibit β-cell function and steroid-induced diabetes is a common side effect of GC treatment. This may be partially mediated by *GAS5*, since GC treatment decreases *GAS5* expression in EndoC-βH1 cells, and both GC treatment and *GAS5* knockdown inhibit GSIS. GC-impaired GSIS was rescued by the overexpression of the active segment of *Gas5* called the hormone response element mimic (HREM), raising the possibility that RNA-based therapeutics could be used to alleviate steroid-induced diabetes [57].

*DANCR* is another lncRNA which acts by sequestering miRNAs, in this case miR-33a-5p which is disproportionally up-regulated in the serum of patients with gestational diabetes. *DANCR* overexpression can rescue the compromising phenotype created by miR-33a-5p overexpression, restoring cell proliferation and insulin output in an INS-1 insulinoma model [58]. Further studies are likely to uncover more lncRNAs that exhibit their function by miRNA sequestration.

## Conclusion

Pancreatic β-cells express over a thousand lncRNAs, although only a small percentage of these have been studied in detail. It is clear from the few which have been investigated that β-cell-specific lncRNAs are regulators of β-cell transcription factor expression, both in *cis* and *trans*, thus regulating β-cell identity. Beyond the transcription factors, lncRNAs have also been shown to regulate expression of other genes, affecting insulin production and secretion, glucose sensitivity, maturity and proliferation.

In a few cases, the molecular mechanisms by which the lncRNAs exert their effects have been revealed, but in many cases they have not. The examples discussed here demonstrate that lncRNAs regulate β-cell gene expression through both chromatin and DNA methylation, affecting chromosomal conformation and both positive and negative effects on miRNA actions. More detailed mechanistic studies of the remaining lncRNAs and distinguishing between direct and indirect gene expression changes will be important to fully understand how lncRNAs regulate β-cell identity and function.

The highly cell-type specific expression of many lncRNAs coupled with their role in regulating β-cell identity raises the possibility that they could be therapeutically targeted to reinforce β-cell function in T2D. lncRNAs also show potential as circulating biomarkers of β-cell dysfunction during T2D.

## Perspectives

- Importance of the Field: Pancreatic β-cell identity is vital for maintaining the patterns of gene expression necessary for correctly regulated insulin secretion. The loss of β-cell identity contributes to type 2 diabetes (T2D) progression, so understanding how cell identity is regulated is central to restoring β-cell function as a new approach to treat the disease. Long non-coding RNAs (lncRNAs) are highly cell-type specific transcripts involved in the regulation of gene expression in a range of tissues.
- Summary of Current Thinking: β-cell specific lncRNAs have been shown to regulate key transcription factors and a variety of other genes underlying β-cell identity and function. lncRNA loci have also been genetically associated with T2D susceptibility, although this appears to be through regulating β-cell mass rather than identity.
- Comment on Future Directions: Given their important role in regulating β-cell identity, the next steps will be to gain greater mechanistic insight into how these lncRNAs function and whether they can be therapeutically manipulated to reinforce β-cell function in T2D. The hundreds of uncharacterised lncRNAs expressed in β-cells may also contain other important players in the complex networks which regulate cell identity.

## Open Access

Open access for this article was enabled by the participation of King's College London in an all-inclusive *Read & Publish* pilot with Portland Press and the Biochemical Society under a transformative agreement with JISC.

## Abbreviations

GC: glucocorticoids
GSIS: glucose-stimulated insulin secretion
GWAS: genome-wide association studies
PRC1: Polycomb Repressive Complex 1
PRC2: Polycomb Repressive Complex 2
SNPs: single nucleotide polymorphisms
